# cSPider – Evaluation of a Free and Open-Source Automated Tool to Analyze Corticomotor Silent Period

**DOI:** 10.1371/journal.pone.0156066

**Published:** 2016-06-01

**Authors:** Skadi Wilke, Dennis Groenveld, Ulrike Grittner, Jonathan List, Agnes Flöel

**Affiliations:** 1 Department of Neurology, Charité-Universitätsmedizin Berlin, Berlin, Germany; 2 Department of Biomedical Engineering, University of Twente, Enschede, Netherlands; 3 Center for Stroke Research Berlin, Charité-Universitätsmedizin Berlin, Berlin, Germany; 4 NeuroCure Cluster of Excellence, Charité-Universitätsmedizin Berlin, Berlin, Germany; 5 Department for Biostatistics and Clinical Epidemiology, Charité-Universitätsmedizin Berlin, Berlin, Germany; University of Ottawa, CANADA

## Abstract

**Background:**

The corticomotor silent period (CSP), as assessed noninvasively by transcranial magnetic stimulation (TMS) in the primary motor cortex, has been found to reflect intracortical inhibitory mechanisms. Analysis of CSP is mostly conducted manually. However, this approach is time-consuming, and comparison of results from different laboratories may be compromised by inter-rater variability in analysis. No open source program for automated analysis is currently available.

**Methods/Results:**

Here, we describe cross-validation with the manual analysis of an in-house written automated tool to assess CSP (*cSPider*). Results from automated routine were compared with results of the manual evaluation. We found high inter-method reliability between automated and manual analysis (p<0.001), and significantly reduced time for CSP analysis (median = 10.3 sec for automated analysis of 10 CSPs vs. median = 270 sec for manual analysis of 10 CSPs). *cSPider* can be downloaded free of charge.

**Conclusion:**

*cSPider* allows automated analysis of CSP in a reliable and time-efficient manner. Use of this open-source tool may help to improve comparison of data from different laboratories.

## 1. Introduction

Transcranial magnetic stimulation (TMS) over the primary motor cortex (M1), while voluntarily contracting the contralateral hand muscle, induces a period of electromyographic (EMG) silence following the motor evoked potential (MEP), that gradually reverts to baseline level. This so called corticomotor silent period (CSP) and its physiology have been the subject of various research studies on inhibitory mechanisms within M1 [1,2]. CSP is assumed to originate from both spinal (early part) and cortical (later part) inhibition [3–6], the latter most likely reflecting intracortical inhibitory mechanisms mediated by gamma aminobutyric acid (GABA)_B_ receptors [7–9].

CSP duration is usually analyzed manually [9–12], which is time-consuming. Moreover, given that it depends on subjective evaluation of onset and offset, inter-rater reliability is poor [13], and therefore compromises between-study comparisons.

Automated software-based approaches may circumvent these problems [13,14], and several laboratories have in fact analyzed their CSP data using such tools, generally as in-house developed routines [13–18]. However, none of these tools is freely available.

Here, we introduce a freely available open-source tool for automated analysis of CSP. We briefly describe the development of the tool, mathematical models underlying its development, and validation of this tool by comparison between manually derived analysis versus automated analysis of CSP datasets from 102 subjects. Time needed for analysis was also noted for each approach.

## 2. Material and Methods

### Subjects

Data of 102 right-handed subjects (32 woman and 70 men, 49 healthy subjects, 53 patients, age range 19–80 years, mean age 44 years) were included in the present study. All subjects fulfilled the following inclusion criteria: 1) No permanent neurological deficit; 2) Normal motor function on neurological examination; 3) No intake of medication that influence the central nervous system; 4) No signs of severe cognitive deficits (Mini Mental State Examination (MMSE) ≥ 26) [19]; 5) No signs of relevant depression (Beck’s depression inventory (BDI) ≤ 12) [20].

We assessed *cSPider* in 53 patients with various neurological diseases including mild cognitive impairment (MCI = 4), mild traumatic brain injury (mTBI = 39), and severe unilateral stenosis or occlusion of the internal carotid artery (ICA = 6) [21]. All these subjects had normal motor functions on routine neurological examination. Structural MRI, as evaluated by an experienced neuroradiologist, revealed no radiologically apparent lesions other than white matter hyperintensities, particularly no territorial stroke or brain tumor.

The study was approved by the local ethics committee of the Charité University Hospital in Berlin/Germany, and performed in accordance with the Declaration of Helsinki. All subjects provided written informed consent.

#### Diagnostics of disease conditions

Diagnosis of MCI was established in the memory clinic of the Charité university hospital by a trained neuropsychologist. Patients fulfilled core clinical criteria for the diagnosis of MCI outlined by Petersen and others [22–24]. Patients reported subjective memory complaints, which were confirmed by standardized neuropsychological testing using the Consortium to Establish a Registry for Alzheimer's Disease test battery (CERAD; Memory Clinic Basel, www.memoryclinic.ch); differential diagnoses were excluded.

Diagnosis of mild TBI required reporting of either confusion for less than 24 hours and/or loss of consciousness for less than 30 minutes following head injury [25].

Patients with severe occlusive process of the ICA (≥80%, according to the European Carotid Surgery Trial (ECST)-criteria) were recruited from the database of our ultrasound laboratory at the Department of Neurology, Charité University Hospital. Unilateral stenosis/occlusion of the ICA was confirmed by extra- and transcranial color-coded Duplex sonography.

### Brain Stimulation

TMS was delivered through a figure-of-eight shaped coil (9 cm outer diameter of each wing), which was connected to a Magstim 200 stimulator (Magstim, Whitland, Dyfed, UK). Participants seated comfortably in a reclining chair. The coil was held tangential to the scalp with the handle pointing backward at an angle of 45° to the interhemispheric fissure. The optimal position (“hot spot”) of the coil was the cortical representation area of the abductor pollicis brevis (APB) muscle (N = 58) or the first dorsal interosseous (FDI) muscle (N = 44) of the contralateral hand. On the “hot spot” a moderately suprathreshold stimulation intensity was leading to visible contraction of the respective muscle of the contralateral hand. The “hot spot” was then marked with a waterproof pen on the scalp of the subject. Motor evoked potentials (MEP) of the respective muscle were recorded via surface electromyographic (EMG) activity using Ag/AgCl surface electrodes in a belly-tendon-montage. Raw MEP signals were amplified and digitized and then stored on a laboratory computer for later offline analysis. The bandpass filter was 5 Hz to 5 kHz (Digitimer). Data were digitized at an analog-to-digital rate of 5 kHz.

#### Resting motor threshold (rMT)

At the “hot spot,” rMT was defined as the stimulus intensity (in % of maximum stimulator output) which was required to produce an MEP of the respective muscle of at least 50 μV in at least five of ten consecutive trials [26].

#### CSP

For this study CSP including preceding MEP were detected. This approach is most widely used in neuroscientific research, and recommended by the IFCN committee [26,27]. CSP was determined for two TMS intensities (120% and 130% of rMT). Participants maintained a voluntary isometric contraction of the respective muscle at approximately 20% of their maximum EMG amplitude, as determined during a maximum voluntary contraction by providing visual feedback from the surface EMG on a computer screen. Ten single-pulse stimulations for each TMS intensity were applied to the “hot spot”. In order to provide a detailed overview for the use of *cSPider* at different TMS intensities, we additionally assessed CSP in a randomly chosen subset of 20 participants (9 women; mean age = 46.8 years, range 19–78 years) at intensities of 110% and 140% of rMT respectively; similar to what had been done by King et al 2006.

### Manual CSP evaluation

**Fig 1:**
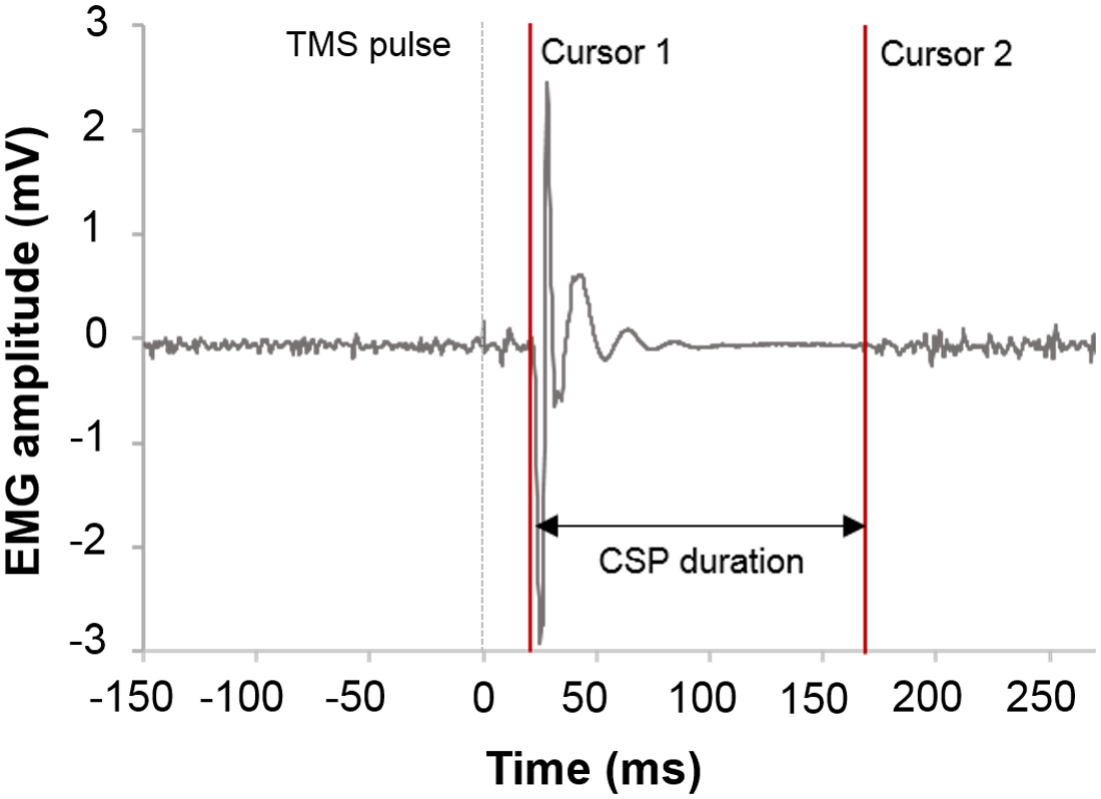
Manual detection of CSP. MEP onset and the subsequent silent period of a single trial are depicted; time from onset of MEP until end of silent period constitute the “CSP duration”. Onset and offset have been marked by setting cursors (red).

For offline analysis Signal software (Cambridge Electronic Design Ltd, Cambridge, UK) was used. Duration of CSP was determined as the time between the MEP onset and the reversion to continuous voluntary EMG activity described as SP2 by van Kuijk et al. (2014) [9]. The CSP onsets and offsets were detected visually and marked manually (Fig 1). Manual analysis was conducted by two different examiners to test for inter-rater variability.

### Automated CSP detection

*cSPider* was used on a PC (3.0 GHz Intel(R) Core 2 Duo CPU, 4.0 GB RAM) running Microsoft Windows 7. Its goal is to automatically detect the CSP onset and offset using the same onset and offset definitions as for the manual detection.

#### Determination of MEP duration

**Fig 2:**
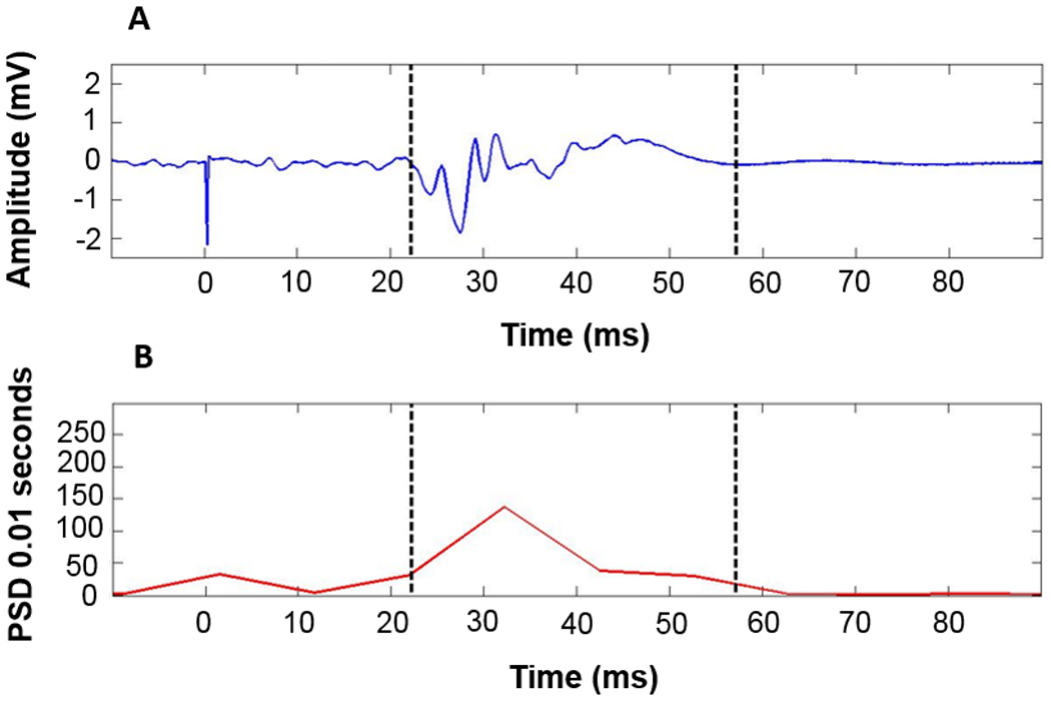
A) Windowed signal for choosing template of a single trial (with estimated MEP onset and offset template). B) Spectrogram for choosing template of a single trial. PDS = power spectral density, mV = millivolt, ms = milliseconds.

The analysis of *cSPider* relies on the spectral power of frequencies over a short time segment. *cSPider* uses spectrograms that divide the EMG frame of interest into n-trial time segments, each with 50% time overlap and windowed with a *Hamming window* in order to sum for all frequencies. The EMG frame of interest is the window used to analyze the CSP and MEP, starting 60 ms before the TMS artifact and ending 420 ms later. In order to determine MEP duration, a user defined template is chosen (black lines Fig 2). Based on this template a matched filter detects similar events across remaining time frames/windows. We here use the filtered peaks above 95% similarity (output matched filter is between 0–1, 1 being 100% similarity) to define the MEP offset based on findings from trial-and-error tests to find a good compromise between the event detection and error (finding of false events, e.g., voluntary contraction) sensitivity.

#### Definition of CSP onset

**Fig 3:**
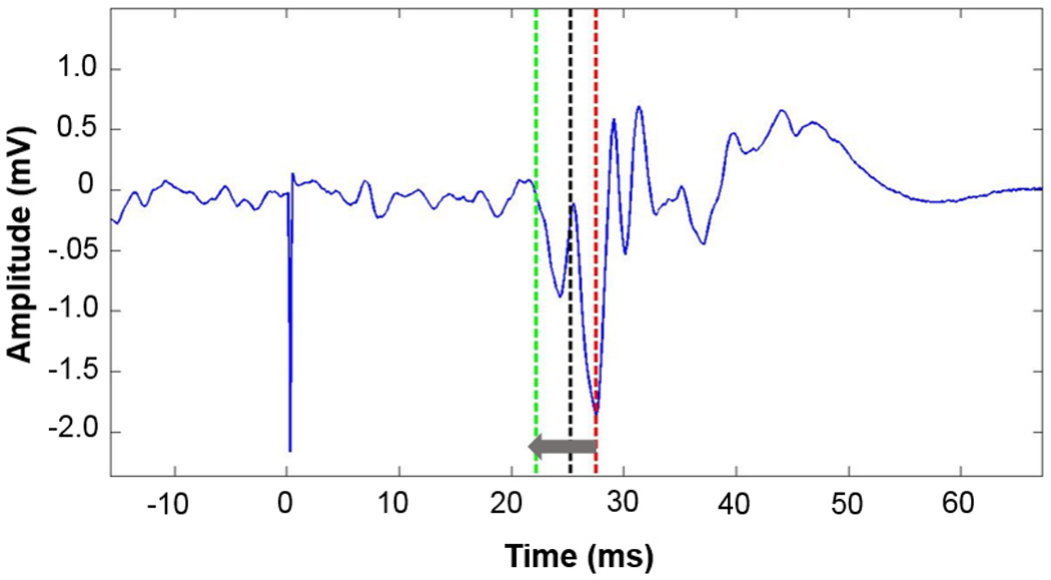
Windowed signal of a single trial. Black: 25 ms past TMS artifact, Red: MEP minimum, Green: CSP onset. mV = millivolt, ms = milliseconds.

To define the CSP onset, the TMS artifact (at time 0 ms in Fig 3) serves as a bench mark. First, the MEP minimum (red line Fig 3) is detected in a window of 40 ms ranging from 5 ms up to 45 ms after the TMS artifact, as it has been shown that the MEP minimum appears around 25 ms downstream from the TMS artifact (black line Fig 3) [27]. Starting from this MEP minimum (red line Fig 3), the search for the CSP onset (green line Fig 3) is conducted within a time window preceding the MEP minimum, as indicated by the grey arrow in Fig 3. The CSP onset is defined as the first data point higher than the averaged negative peaks from the pre-pulse EMG data. For this purpose the average of all negative peaks between the current and the previous EMG frame of interest is used. Daskalakis et al. (2003) reported a similar approach; however, instead of averaging only negative peaks, they used the mean of the rectified pre-pulse EMG and defined the CSP onset as the first value that crosses the averaged pre-pulse [17]. Note that the *cSPider* approach does not necessitate rectification of data.

With *cSPider* a sufficient range of negative peaks from the pre-pulse EMG data is considered in order to enable a consistent determination of CSP onset.

#### Definition of CSP offset

**Fig 4:**
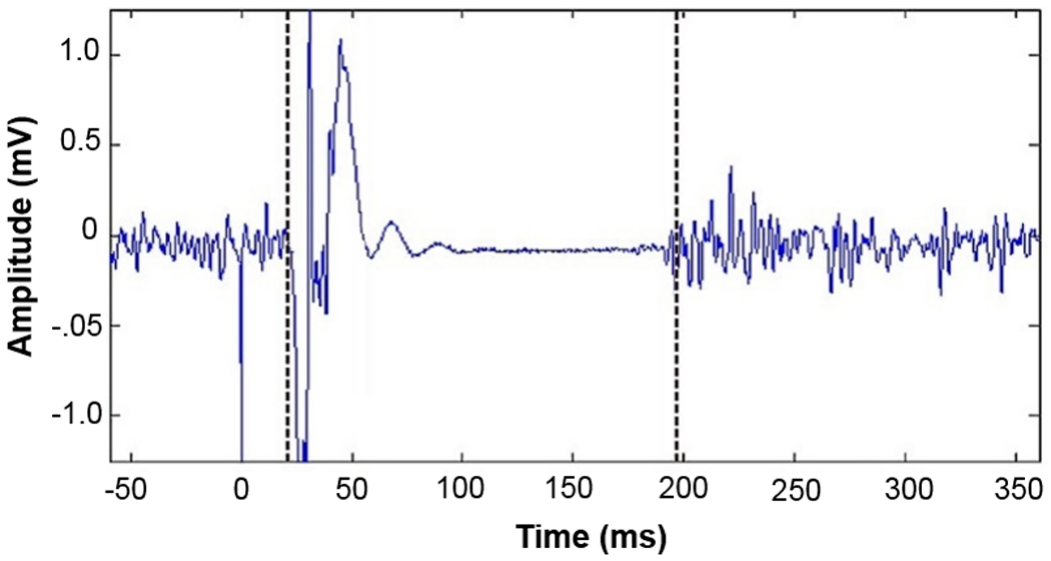
Example window—found CSP onset and offset of a single trial are depicted. mV = millivolt, ms = milliseconds.

A search window, ranging from 10–400 ms after MEP offset (automatically detected), is determined by *cSPider*. Within this window, only absolute derivative of the high-pass filtered data (cut-off frequency = 0.016) are taken into account. Subsequently, the first data point of the moving average (= average taken over 30 data points) exceeding 75% of the average taken over the absolute derivative of the high-pass filtered pre-pulse EMG data (with same cut-off frequency) is defined as the CSP offset. This point equals the return to continuous voluntary EMG activity (Fig 4).

To exclude false positive CSP, thresholds for highly unlikely CSP duration have been defined (cut-off ≤ MEP + 5ms). If a CSP duration exceeds this threshold a search window will open for visual control. This option allows either manual correction of the respective CSP measurement, or discarding of the measurement. For the purpose of this paper, all participants where CSP durations exceeded this threshold were discarded to evaluate the quality of automated CSP determination only.

#### Automated CSP measurement– 2 further options for determining MEP duration

The second approach consisted of a low-pass filter with the same cut-off frequency of 0.016 combined with finding the second peak of minimal 2 mV of its absolute, making use of the typical low frequency behavior which in most cases consisted of a clear slow sinusoid of 1 wavelength and high amplitude. However, because not all patients showed these typical EMG forms, this method should not be used and only taken into account for a better insight and a possible future combination of methods for the analysis of MEPs. The third and last option considered a fixed MEP duration of 25 ms. This approach allows for fast analysis. However, then the option to take into account subject specific MEP durations is lost.

#### Time needed for manual and automated approach

Time for manual and automated determination of 10 CSPs was recorded for the same subset of 21 subjects (i.e., 420 single CSP) which were chosen randomly. As it is a default setting in *cSPider* that the resulting CSP durations are automatically stored in an extra data file, time recording for the manual approach started with marking CSP onset and offset and stopped after copying CSP durations in a data file. For the automated approach (with and without visual feedback) time recording started with choosing the dataset and stopped when the resulting CSP durations were stored automatically in an extra data file; see also Fig 4.

## 3. Statistical Analysis

We used SPSS Statistics 22.0 (IBM Corporation, Armonk, NY). Normal distribution of data was checked via histogram and skewness (absolute skewness<1). Two-sided alpha level was set to 0.05 for all statistical analyses.

### Inter-rater and inter-method reliability

First, the inter-rater agreement between the two examiners was computed using intra-class correlation (ICC). Second, an ICC for the manual and automated method was conducted using the mean of the two manually detected CSP duration. To determine the ICC a two-way mixed model with absolute agreement was conducted [28]. In addition, the agreement between rater 1 and rater 2, and manual and automated approach respectively were depicted as previously reported by Bland and Altman [29]. The limits of agreement were defined as mean difference ± 1.96 SD of difference.

### Comparison of time for analysis in manual vs automated approach

A histogram of the variable durations for analysis indicated a deviation from normal distribution (absolute skewness>1). Thus, a nonparametric Wilcoxon signed rank test for related samples was used to compare time needed for manual detection of CSP with time needed for automated detection (with and without visual feedback).

## 4. Results

### Automated CSP

11 datasets were discarded from further analysis, as CSP duration exceeded the threshold (cut-off≤MEP+5ms) due to low data quality caused by artifacts, or failure to evoke MEP and thus CSP. Overall, 15 CSPs out of these 11 datasets required manual correction. After excluding these datasets, *cSPider* was able to identify 98.5% of all manually detected values correctly as CSP. Moreover, *cSPider* showed a false-positive rate of 1.32%.

### Inter-rater reliability (ICC)

**Fig 5:**
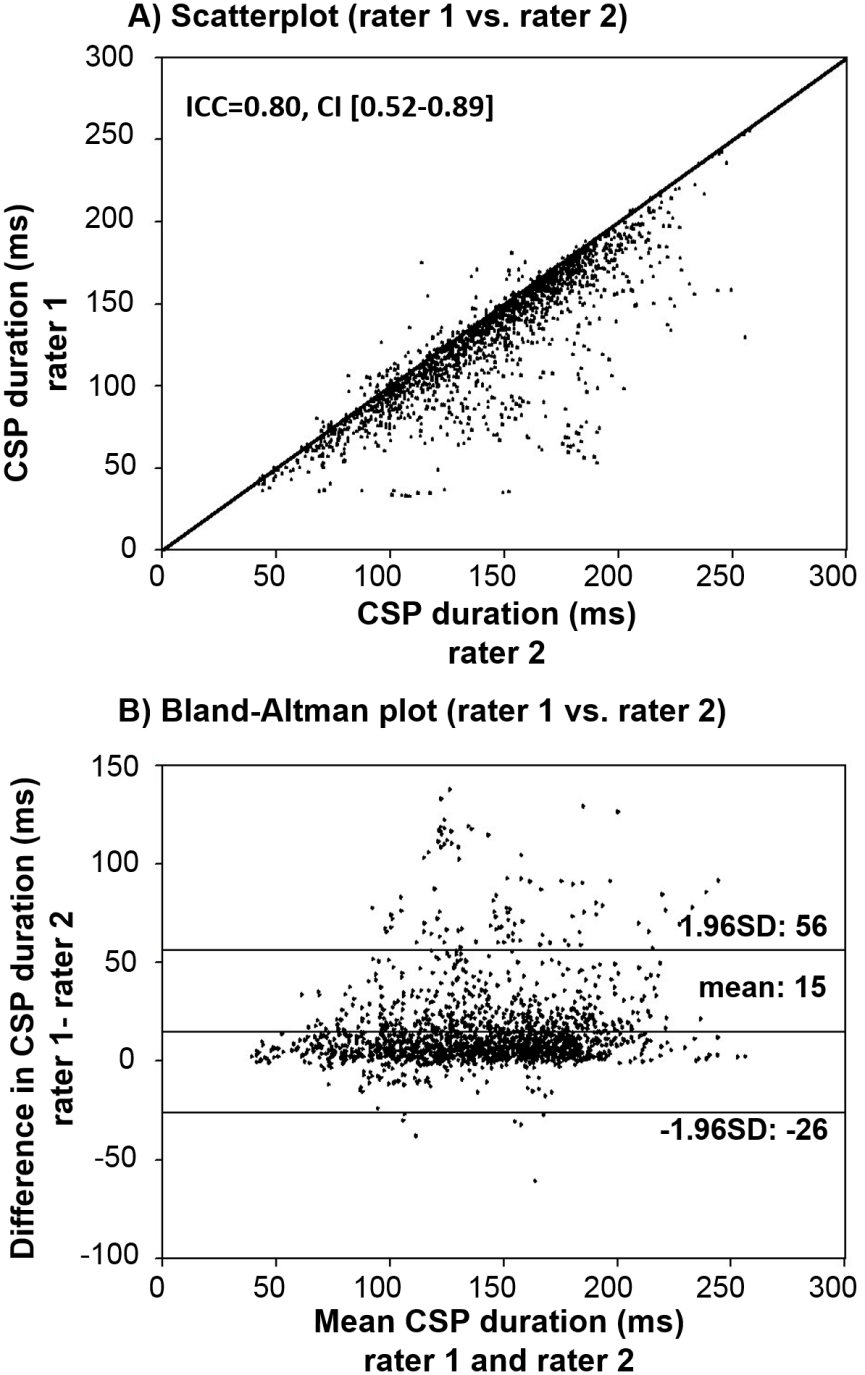
(A) Scatter-plot between the manually detected CSP durations at 120% and 130% of rMT. A line of identity (y = x) is integrated; ICC = 0.86 and confidence interval (CI) = [0.83–0.88], indicating a high inter-method agreement between the manual and the automated approach. (B) Bland–Altman plot [29]. Upper and lower lines represent the limits of agreement (mean±1.96 SD), ms = milliseconds.

Comparing the two manual analyses, the single measure ICC was 0.80 with a 95% confidence interval from 0.52 to 0.89 (Fig 5). Table 1 provides a detailed overview of group specific ICC. The limits of agreement between rater 1 and rater 2 were -26-56 ms (Fig 5B).

**Table 1:** Inter-rater- and inter-method-agreement (ICC) for the different subject groups (without discarded CSP duration).

Group	Comparison	ICC [95% CI]	Number of CSP
Healthy	Rater 1 vs. Rater 2	0.82 [0.72–0.88]	941
(N = 50)	Manual vs. *cSPider*	0.80 [0.76–0.83]	954
TBI	Rater 1 vs. Rater 2	0.95 [0.90–0.97]	648
(N = 33)	Manual vs. *cSPider*	0.93 [0.91–0.94]	660
MCI	Rater 1 vs. Rater 2	0.86 [0.60–0.94]	80
(N = 4)	Manual vs. *cSPider*	0.89 [0.56–0.95]	80
ICA	Rater 1 vs. Rater 2	0.97 [0.96–0.98]	80
(N = 4)	Manual vs. *cSPider*	0.80 [0.70–0.87]	80

N = number of participants, CSP = corticomotor silent period, ICC = intraclass correlation coefficient; CI = Confidence interval; df = degrees of freedom; Healthy = Healthy subjects; TBI = traumatic brain injury; MCI = mild cognitive impairment; ICA = internal carotid artery.

### Inter-method reliability (ICC)

**Fig 6:**
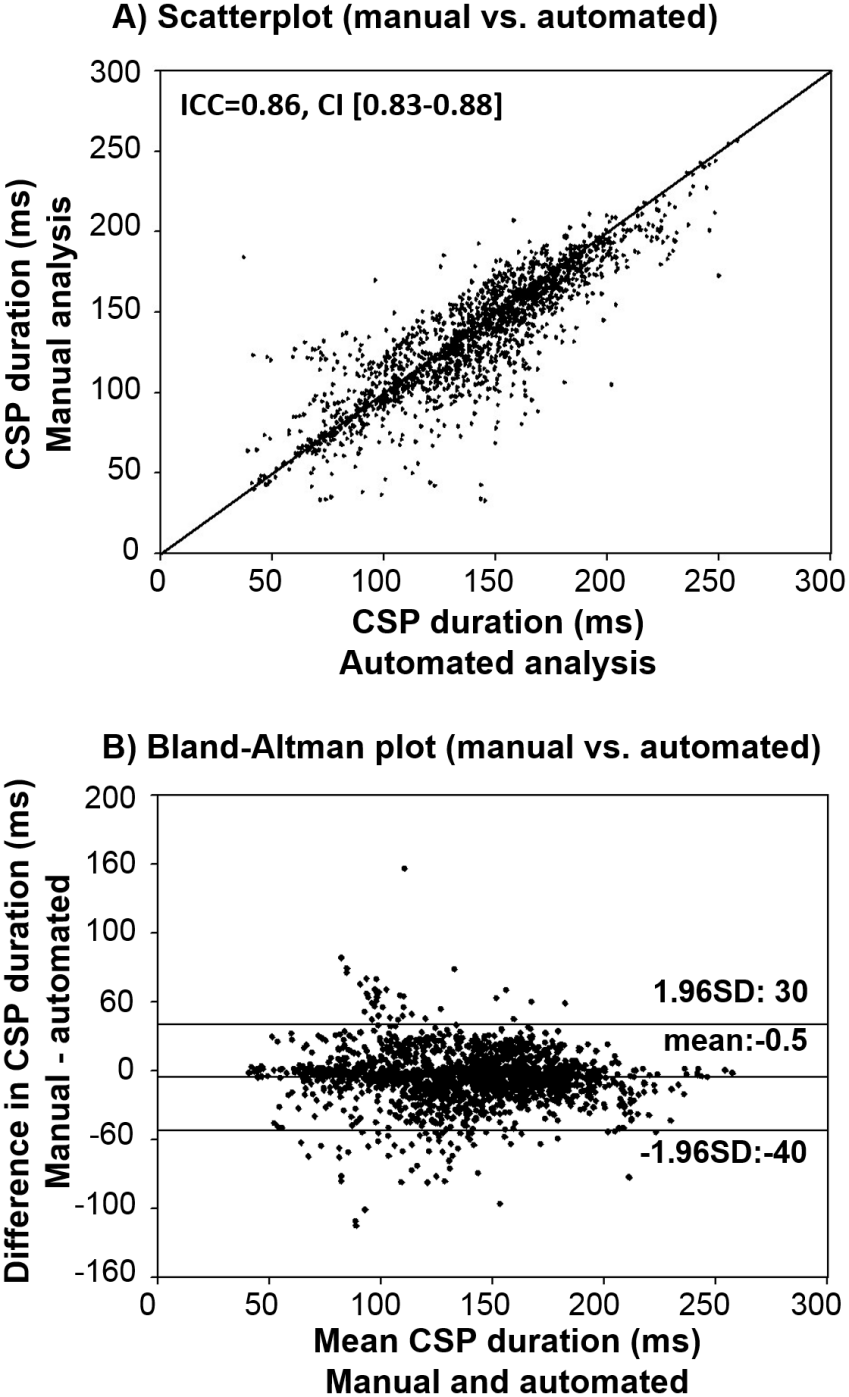
(A) Scatter-plot between the automated and manual analysis of CSP durations at 120% and 130% of rMT. A line of identity (y = x) is integrated; ICC = 0.86 and confidence interval (CI) = [0.83–0.88], indicating a high inter-method agreement between the manual and the automated approach. (B) Bland–Altman plot [29]. Upper and lower lines represent the limits of agreement (mean±1.96 SD), ms = milliseconds.

Comparing the automated and manual approach at intensities of 120% and 130% a high degree of reliability was found. The single measure ICC was 0.86 with a 95% confidence interval from 0.83 to 0.88 (Fig 6). The limits of agreement between the manual and automated approach were -40-30 ms (Fig 6B).

**Fig 7:**
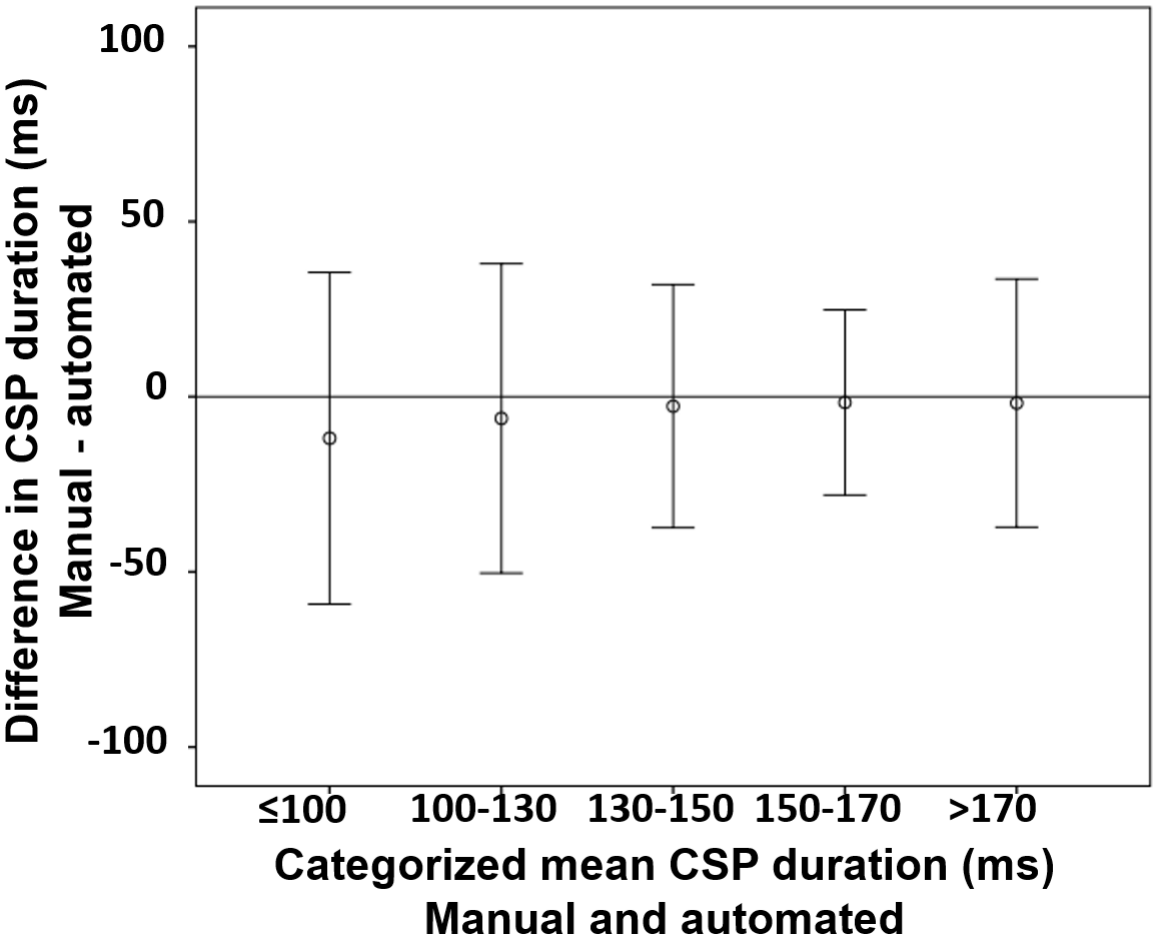
Mean difference in automatically and manually detected CSP durations categorized by the mean CSP duration, Error bars equal 2 SD, ms = milliseconds.

Furthermore, to address the question if non-systematic differences for the CSP detection occur at lower stimulus intensities, a figure was inserted that depicts an error bar plot, showing the variance in the difference of manually and automatically detected CSP durations grouped by the size of mean CSP durations (Fig 7).

The depicted error bars represent 2 SD and thus show the region in which 95% of the data lies. Differences between manually and automatically detected CSPs are similar in the different categories, except for the category of CSP ranging between 150 and 170 ms that shows a smaller range of differences (manual vs. automated).

### Inter-method reliability for low and high stimulus intensities

Comparison of automated vs manual CSP detection for a subset of participants (N = 20) at 110% of rMT and 140% of rMT yielded a slightly lower reliability for low intensities (110%), as compared to higher intensities (140%): For 110% of rMT, single measure ICC was 0.76 with a 95% confidence interval from 0.55 to 0.86; for 140% of rMT, the single measure ICC was 0.84 with a 95% confidence interval from 0.55 to 0.93. For a detailed overview of intensity specific ICC see Tables 1–3.

**Table 2:** Inter-rater- and inter-method-agreement (ICC) for different TMS intensities analyzed for a subset of 20 subjects.

TMS intensity	Comparison	ICC [95% CI]	Number of CSP
110%	Rater 1 vs. Rater 2	0.75 [0.59–0.84]	192
(N = 20)	Manual vs. *cSPider*	0.76 [0.55–0.86]	194
120%(N = 20)	Rater 1 vs. Rater 2	0.69 [0.58–0.77]	200
(N = 20)	Manual vs. *cSPider*	0.75 [0.47–0.86]	200
130%	Rater 1 vs. Rater 2	0.73 [0.60–0.81]	200
(N = 20)	Manual vs. *cSPider*	0.81 [0.53–0.90]	200
140%	Rater 1 vs. Rater 2	0.85 [0.72–0.91]	200
(N = 20)	Manual vs. *cSPider*	0.84 [0.55–0.93]	200

N = number of participants, CSP = corticomotor silent period, ICC = intraclass correlation coefficient; CI = Confidence interval; df = degrees of freedom.

**Table 3:** Inter-rater- and inter-method-agreement (ICC) for different TMS intensities analyzed for 91 subjects (without discarded CSP duration).

TMS intensity	Comparison	ICC [95% CI]	Number of CSP
120%	Rater 1 vs. Rater 2	0.77 [0.48–0.88]	880
(N = 91)	Manual vs. *cSPider*	0.83 [0.79–0.86]	886
130%	Rater 1 vs. Rater 2	0.80 [0.52–0.89]	890
(N = 91)	Manual vs. *cSPider*	0.87 [0.84–0.88]	880

N = number of participants, CSP = corticomotor silent period, ICC = intraclass correlation coefficient; CI = Confidence interval; df = degrees of freedom.

### Time to analyze CSP manually vs automated (*cSPider*)

Wilcoxon signed-ranks test indicated that the automated approach without visual feedback (median = 10.3 sec, Z = -4.02, p<0.001, r = -0.08) and with visual feedback (median = 61.1 sec, Z = -4.02, p<0.001, r = 0.04) were significantly less time-consuming than the manual analysis (median = 270 sec). Thus, using *cSPider* without the option of visual feedback for N = 30 subjects (20 trials per subject) resulted in an average time saving of 45.3 hours.

## 5. Discussion

We here introduce a novel software to automatically analyze CSP, termed *cSPider*, available open-source and free of charge. We demonstrate that *cSPider*, compared to manual analysis, reliably detects CSP derived from different TMS intensities in healthy subjects and patients with various neurological diseases, with significantly higher speed. Additional analyses regarding the differences between manual and automated CSP detection show no non-systematic variance for different stimulation intensities.

### CSP in neuroscientific and neurological research

CSP is a widely used protocol to investigate GABA_B_ activity in human M1 [30–32], and has revealed important insights into cortex physiology in health and disease [33–35]. For example, differences between patient populations and controls have been reported for epilepsy [36,37] and traumatic brain injury [11,38]. Moreover, CSP has been useful to investigate changes in intracortical inhibition as induced by pharmacotherapy in healthy controls [39]. Moreover, CSP can evaluate the mechanisms underlying changes in corticomotor excitability and behavior induced by transcranial direct current stimulation [40,41], which may be due to shifts in cortical GABA_B_ activity [40].

### Automated CSP tools and comparison with *cSPider*

Several previous studies reported the implementation of in-house written automated routines to analyze CSP [13–15,17,42]. The aim of *cSPider* and most of these approaches is the signal detection; note though that the routine of Rábago et al. (2009) additionally aims to model the CSP. Nilsson et al. used a combined graphical and mathematical approach by plotting the data logarithmically and subsequently conducting Student’s t tests to solve the problem of automated CSP detection. The tool by Daskalakis et al. (2003) used a rather mathematical approach by automatically processing pre-stimulus EMG activity to define a threshold for MEP onset detection which equaled the CSP onset. In contrast to these approaches it is not necessary to transform data files into ASCII format or any other data format for *cSPider*.

Recently Julkunen et al. (2014) developed a tool for online CSP detection which does not depend on pre- or post-CSP EMG levels. Similar to the automated routines of Daskalakis et al. (2003), King et al. (2006) and Nilsson et al. (1998) *cSPider* also relies on the processing of pre-pulse EMG activity. Julkunen et al. (2014) argue that muscle contraction has little effect on CSP duration. But as pre-pulse EMG activity is used in most of the automated routines and reliable CSP detection has been reported with this approach [13,14,17,43], we decided to use pre-pulse EMG activity for *cSPider* as well.

In line with Daskalakis et al., Garvey et al. (2001) and Julkunen et al., *cSPider* uses the difference between consecutive data points for CSP detection.

In addition, subject-specific MEP durations, rather than fixed MEP durations, are calculated by *cSPider*, aiming to generate more exact CSP durations. We also implemented the option to visually inspect automated CSP detection in order to control quality of this detection. In addition, a graphical user interface has now been implemented to improve the usability of *cSPider*, a feature not available in approaches.

Note though that Julkunen et al. (2014) reported the highest reliability coefficients for their automated routine, with excellent (low) limits of agreement. However, their approach is not open-source. The other automated approaches did not report limits of agreement, thus a comparison of *cSPider* on the basis of limits of agreement was not possible. With the present study and paper, we provide our source code to the open-source community and following the “bazaar model” [44] aim to initiate a cooperation between research groups and experts to detect weaknesses over time, and subsequently to improve on the code.

### *cSpider* is an open-access product

As already pointed out, *cSPider* offers the advantage that it is an open-source tool, i.e., can be downloaded and used free of charge. As the number of experimental and clinical studies using CSP is high, there is a growing necessity for freely accessible automated tools. As sharing key components of data from publications is currently discussed and increasingly called for by researchers of different disciplines and journals [45], we hope that making this well-described and validated tool freely available will help improve standardization [46], reproducibility [47] and comparability [46] of results within and between research groups.

### *cSPider* is user-friendly and saves time

*cSPider* was developed for researchers starting in the field of neurophysiology, i.e., master or graduate students, or for routine clinical use. We therefore implemented a user-friendly GUI and furthermore provide a basic and easy-to-understand user guide, downloadable with the tool, to improve usability and to allow for its use with only minimal previous programming knowledge. In addition, the open source code allows advanced researchers to customize parameters and even to improve on the code within the *cSPider* protocol. Significant time savings for analyses (i.e., 45 hours for a dataset of N = 30 subjects) will be appreciated both in the clinical and the research environment.

### Limitations

First, *cSPider* relies on a Matlab^®^ environment, necessitating the use of a comparatively expensive program. However, most neuroscience research labs and even clinical platforms use Matlab^®^ for various purposes already. Second, *cSPider* detects CSP duration which includes the preceding MEP; the detection of silent period only has not yet been implemented. Note though that the CSP including preceding MEP has been proposed by the most recent report from the IFCN committee [48], and thus may be considered most appropriate. Third, so far *cSPider* is not able to detect CSP without preceding MEP, implemented for example in the automated routine of King et al. (2006) and Rábago et al. (2009). However, given that our laboratory only uses CSP protocols with stimulus intensities of at least 110% of rMT, a preceding MEP is found consistently [47]. In the future, the tool may be developed to also include detection of CSPs without preceding MEP.

Fourth, no data for specific CSP onset and offset times have been collected as it has been done in a study of Daskalakis et al. (2004). Further analyses of these parameters could be done in order to provide detailed information about the quality of *cSPider* as it has been shown that there could be discrepancies between ICC for CSP duration and ICC for onset and offset times [17].

Fifth, as indicated by the Bland-Altman plot (Fig 6) and the error bar graph (Fig 7) there is still variation in the difference between manual and automated analysis; thus, the algorithm should be improved in the future within the open-source project.

Furthermore, we used CSP data derived from two different muscles—FDI and APB muscle—which should not affect the comparison of the manual and automated approach, given that previous studies indicated that CSP duration from these muscles are highly correlated [49].

Considering these points, it is explicitly appreciated to work on these issues in order to detect weaknesses and improve the code by the open-source community, with the aim to validate *cSPider* by other research groups in the fields.

## 6. Conclusion

*cSPider* allows for automated analysis of CSP in a reliable and time-efficient manner. Use of this open-source tool may help to improve comparison of data from different studies and across laboratories. We highly encourage other researchers to use and improve *cSPider*, as it is the first automated method which is shared online free of charge.
